# Sexual, Reproductive Health Needs, and Rights of Young People in Slum Areas of Kampala, Uganda: A Cross Sectional Study

**DOI:** 10.1371/journal.pone.0169721

**Published:** 2017-01-20

**Authors:** Andre M. N. Renzaho, Joseph K. Kamara, Nichole Georgeou, Gilbert Kamanga

**Affiliations:** 1 Humanitarian and Development Research Initiative, School of Social Sciences and Psychology, Western Sydney University, Sydney, NSW, Australia; 2 Department of Epidemiology and Preventive Medicine, Monash University, Melbourne, Victoria, Australia; 3 World Vision International, Southern Africa Regional Office, Mbabane, Hhohho, Swaziland; 4 World Vision Uganda, Kampala, Kampala City, Uganda; National Institute of Health, ITALY

## Abstract

**Background:**

Young people in Uganda face various sexual and reproductive health risks, especially those living in urban slums. The aim of this study was to examine factors associated with comprehensive categories of sexual and reproductive health, including sexual behaviours; sexual education and access to contraceptive services; family planning; prevention of STDs; sexual consent as a right; gender based violence; as well as HIV testing, counselling, disclosure and support.

**Methods:**

The study was cross-sectional in design and was carried out in July 2014 in Makindye and Nakawa Divisions of Kampala City, Uganda. Using systematic random sampling, data were collected on 663 participants aged between 13 and 24 years in Kampala’s urban slums.

**Results:**

Sixty two percent of participants reported having ever had sex and the mean age of sexual debut was 16 years (95%CI: 15.6, 16.4 years, range: 5–23 years). The odds of reporting ever having had sexual intercourse were higher among respondents living alone (OR: 2.75; 95%CI: 1.35, 5.61; p<0.01) than those living in a nuclear family. However, condom use was only 54%. The number of sexual partners in the last 12 months preceding the survey averaged 1.8 partners (95%CI: 1.7, 1.9; range 1–4) with 18.1% reporting an age gap of 10 years or older. More than three quarters (80.6%) of sexually active participants reported that their first sexual encounter was consensual, suggesting that most young people are choosing when they make their sexual debut. Low prevalence of willing first sexual intercourse was associated with younger age (OR = 0.48, 95%CI: 0.25, 0.90, p<0.05), having a disability (OR = 0.40, 95%CI: 0.16, 0.98, p<0.05), living with non-relatives (OR = 0.44, 95%CI: 0.16, 0.97, p<0.05), and being still at school (OR = 0.29, 95%CI: 0.12, 0.67, p<0.01). These results remained significant after adjusting for covariates, except for disability and the age of participants. The proportion of unwilling first sexual intercourse was significantly higher among women for persuasion (13.2% vs. 2.4%, p<0.001), being tricked (7.1% vs 2.9%, p<0.05) and being forced or raped (9.9% vs 4.4%, p<0.05) than men. A high level of sexual abuse emerged from the data with 34.3% affirming that it was alright for a boy to force a girl to have sex if he had feelings for her; 73.3% affirming that it was common for strangers and relatives to force young females to have sexual intercourse with them without consent; 26.3% indicating that it was sometimes justifiable for a boy to hit his girlfriend, as long as they loved each other.

**Conclusion:**

This study has explored current sexual practice among young people in a specific part of urban Kampala. Young people’s sexual and reproductive health remains a challenge in Uganda. To address these barriers, a comprehensive and harmonised sexual and reproductive health system that is youth friendly and takes into account local socio-cultural contexts is urgently needed.

## Introduction

Sexual and reproductive health and rights (SRHR) broadly applies the concept of human rights to sexuality and reproduction, and is concerned with the intersection of four distinct fields—sexual health, sexual rights, reproductive health, and reproductive rights—of sexual and reproductive wellbeing [1, 2]. To maintain one’s sexual and reproductive health, access to accurate information (e.g. seek, receive, and impart information related to sexuality) and a choice of safe, effective, affordable contraception options are key [2, 3]. A human rights framework emphasizes access to information to empower individual freedom of choice with respect to: deciding whether to be sexually active or not (e.g. sexual debut); the pursuit of a satisfying, safe, and pleasurable sexual life; choosing a partner; consensual sexual relations and consensual marriage; protection from sexually transmitted infections (STIs); and family planning (e.g. whether or not, and when, to have children) [2]. The availability of, and access to, health and information services for women and girls that facilitate healthy pregnancies, births, mothers and babies is also essential to good sexual and reproductive health [4].

Young people in sub-Saharan Africa face various sexual and reproductive health risks such as unplanned pregnancy and sexually-transmitted infections (STIs), including HIV [5, 6]. Sub-Saharan Africa has a high level of HIV, with the 2015 Global Health Observatory data suggesting that the region remains most severely affected, and has the highest adult HIV prevalence [7]. Compared with an average HIV prevalence of 0.8% among adults aged 15–49 years worldwide, nearly one in every 25 sub-Saharan African adults (4.4%) live with HIV, and the region accounts for nearly 70% of the people living with HIV worldwide [7]. With an HIV prevalence of 4.4%, sub-Saharan Africa compares unfavourably with South East Asia (0.3%), the Americas (0.5%), Europe (0.4%), East Mediterranean (0.1%) and the Western Pacific (0.1%) [7]. Similarly, other studies in sub-Saharan Africa have shown a high prevalence of syphilis, gonorrhoea, bacterial vaginosis, trichomoniasis and herpes simplex virus type 2 [8, 9].

In the last decades of the 20^th^ century Uganda achieved tremendous progress in fighting the HIV/AIDS pandemic, and reduced the prevalence of the disease in the general population from 15% in the early 1990s to 7.0% in 2014 [10]. However, HIV/AIDS in Uganda is unequally distributed and some sections of the population are more affected than others, with the prevalence higher in urban areas than rural areas (8.7% vs. 7%), higher in women than men (8.3% vs. 6.1%), and two-to-three times higher among fishing communities (22%), commercial sex workers (35%) and men who have sex with men (13%) [10]. Among young people aged 15–24 years, the prevalence of HIV/AIDS is significantly higher among young women (4.2%) than young men (2.4%) [10]. In addition, overall around one in three young people have comprehensive and correct knowledge of HIV/AIDS [10]. Furthermore, Uganda retains a high burden of other sexual and reproductive health risks such as teenage pregnancy, with 33% of Ugandan women having given birth before the age of 18 years which is among the highest in the world [11]. The high pregnancy rate among adolescents is compounded by the unmet contraceptive needs of almost half of the fertile and sexually active women [12].

Whilst Uganda has a national adolescent health policy that aims to streamline adolescent health concerns into the national development process to improve young people’s quality of life and standard of living [13], its impact on the sexual and reproductive health needs of young people has been less than adequate. For example, previous studies [14–16] have identified young people in Uganda and other sub-Saharan African countries as having limited access to contraception, and a lack of staff trained to address the sexual health needs and education gaps of young people. Key issues that have negatively impacted upon young people’s sexual and reproductive health include unwanted pregnancies, sexually transmitted infections (STIs), defilement, rape, and substance abuse [14]. Access to legal abortion also poses a challenge as it is legally restricted [17–20].

Whilst it has been well documented that recognising adolescents and young people as a priority is one of the prerequisites to achieving bold international goals such as the millennium and sustainable development goals [21, 22], adolescent health, especially sexual and reproductive health, remains a challenge and is poorly addressed by these international development agendas [23]. Suboptimal sexual and reproductive health not only increases morbidity, mortality, and gender inequity, it also slows development [14, 24]. Addressing adolescent sexual and reproductive health needs in Uganda is especially important, as a growing number of young people are sexually active [14]. There is a strong link between sexual abuse and the risk of unintended pregnancy [25]. Coerced sexual debut has been found to be associated with increased risk of ongoing coercion, which has adverse reproductive health outcomes including: decreased contraceptive use; non-use and inconsistent use of condoms; unintended pregnancy; and genital tract symptoms, possibly indicating the presence of an STI [25]. Early marriage and early sexual activity often drive a high incidence of complications from pregnancy and delivery. In addition, sexual advances by older men and practices of transactional sex make adolescent females particularly vulnerable to sexual, reproductive and health risks [26].

In Uganda, there is a discrepancy between universally formulated sexual and reproductive health rights and the local political, economic and community contexts in which young people live [27]. This discrepancy requires that a comprehensive rights-based sex education takes this local reality into consideration [27], however, most of the studies on sexual and reproductive health rights summarised above focused on the limited dimensions of sexual health rights (SHR). Specifically they are narrow in scope and are not comprehensive in the analysis of the local socio-cultural contexts that shape the sexual and reproductive health needs and rights of young people in Uganda. To comprehensively explore the socio-cultural contexts that influence sexual and reproductive health needs and rights of young people in Uganda, the application of a societal theory is indispensable. The socio-ecological theory was adopted in this study to explore the interdependent complex relationships that exist between individual (i.e. skills, knowledge, attitudes, beliefs and behaviours), interpersonal (i.e. social networks and social support systems that influence individual behaviours, including family, friends, and customs or traditions), community (i.e. established norms and values including rigid gender roles, peer pressure, loss of traditional support, and economic hardships), organizational (i.e. rules and practices such as disapproval from health workers, geographical accessibility, inadequate counselling from health workers or lack of privacy), and environment (i.e. policies on sexual and reproductive health, HIV, and AIDS including HIV/AIDS counselling), factors [28, 29].

In addition, while HIV prevalence is typically higher in urban than in rural settings in sub-Saharan Africa there are strong intra-urban differences in the risk of HIV infections. While a growing body of research has focused on poor health outcomes among the urban poor, HIV and its risk factors have attracted little attention. The low employment opportunities outside the capital means that Kampala attracts a large numbers of young people in search of work. Within Kampala, the two divisions of Nakawa and Makindye is where the research project was carried out. Over the past two decades these two divisions have been characterised by high unemployment in concert with a high dependence burden and rampant crime, which presents strong barriers to youth participation in community programmes and negatively affects their well-being [30]. Despite the dire economic and social situations that predispose youth to HIV, studies into their sexual behaviours, sexual education and access to contraceptive services, family planning, sexual consent as a right, and gender based violence are lacking. The few available data emerging from Kenya suggest that residents of urban slum areas have a higher HIV prevalence than urban non-slum residents and engage in riskier sexual practices than other sub-groups [31]. Therefore, the aim of this study was to examine factors associated with comprehensive categories of sexual and reproductive health among young people in slum areas of Kampala, Uganda, including: sexual behaviours; sexual education and access to contraceptive services; family planning; prevention of STDs; sexual consent as a right; gender based violence; as well as HIV testing, counselling, disclosure and support.

## Methods

### Study design and sampling strategies

The study was cross-sectional in design and was carried out in July 2014 in Makindye and Nakawa divisions of Kampala City, Uganda. The study focused on youth (13–17 years) and young adults (18–24 years). Uganda has one of the youngest and fastest growing populations in Africa, with a 2013 estimate placing it at 35.5 million. More than three quarters (78%) are aged below 30 years [32]. Ugandan youth make up the largest proportion of the population, but they are negatively affected by youth unemployment rates of 61.6%, among the highest in Africa [33, 34]. The absence of employment opportunities outside of the capital Kampala has led to significant numbers of young people entering the city in search of work.

The Kampala City Council is composed of divisions, and each administrative division is comprised of parishes, divided into zones. A total of 391 zones were identified in the two target divisions. A list of all zones within the parishes of the Makindye and Nakawa divisions was established for sampling purposes. A list of households in each of the zones was constructed with the help of the local councils, and from the list, a household was selected using a systematic sampling approach. Households in each zone were given a unique identification number. Given that zones varied in size, the number of households to be surveyed in each zone was proportional to the size of the zone. The sampling interval (X) was determined by dividing the total number of households in each zone with the expected sample size, and the first household to be surveyed was randomly selected by choosing a number between 1 and X. The next household to be visited was selected by adding X to the first randomly selected number, and the process continued until the required sample size for that zone was obtained. For each selected household, a person aged 13–24 years volunteered to take part in the study and the interview occurred outside the home, away from other household members. If the selected household was not inhabited, or there was no one at home, the closest neighbouring household was used for the survey. In the case of a household having more than one eligible participant, the interviewer randomly selected one participant to be included in the study. The study was approved by the Monash University Human Research Ethics Committee (approval no. CF16/1001-2016000532).

### Procedure and data collection

Studies in sub-Saharan Africa have shown that low community awareness about health research and the participants’ perception that signing consent forms is not a good approach due to the potential legal accountability decrease research acceptance and participation rates [35]. Therefore, trained data enumerators, who were bilingual (English and Luganda) explained the study to participants in either English or Luganda through a plain language statement. Written and verbal consent (witnessed) was sought from participants. For children aged 13–17 years we did not seek parental permission for two reasons: 1) children were able to understand or appreciate what the research entailed given their exposure to multi-media HIV campaigns at all levels of community structures [36], and 2) discussing sexual matters was a sensitive issue and seeking parental consent would have been inappropriate and may have prevented young people discussing freely their sexual health needs and rights [37]. Where participants were able to provide a signature, signed informed consent was secured. For those who could not sign due to cultural and political reasons, the consent was oral. Since data collectors worked in pairs due to security reasons, the oral consent was recorded by the primary data interviewer on behalf of the participant and witnessed by the assisting data collector [38].

A total of 834 households were contacted, of which 158 did not participate due to family commitments; a further 13 interviews from 13 households were incomplete and were excluded from the analysis, giving a total sample size of 663 interviews with valid data and a response rate of 79.5%. Data were collected by 12 trained enumerators, who were supervised by four experienced field coordinators to monitor quality control. Data enumerators, who were bilingual (English and Luganda) were trained over three days, followed by a field testing of the questionnaire prior to data collection to ascertain its cultural appropriateness. The training covered sampling techniques, interview techniques and ethical issues including confidentiality and respect (i.e. the right of a participant to refuse to respond to a question or to the entire survey), and participants’ familiarisation with the questionnaire. Bilingual workers administered the survey in English. The research implementation was overseen by a steering committee comprised of staff from World Vision, four field coordinators, and representatives from youth organisations. The steering committee approved all processes and commented on the questionnaire.

### Survey instruments

A structured questionnaire was used to collect data at the household level. It had four sections: demographics of household members (age, gender, ethnic group, educational attainment and religion), sexual behaviours, sexual and reproductive health rights, and sexual and reproductive health needs. The questionnaire was field tested prior to data collection for cultural appropriateness and clarity and was administered in English by trained bilingual workers, translating into Luganda when appropriate.

### Statistical analysis

Data were analysed using Stata. In the first instance, a descriptive univariate analysis was undertaken. Then the relationship between two categorical variables (i.e. study outcome versus independent variable) was initially examined using the chi-square test. Adjusted logistic regression analyses were performed to determine the best prediction of a dependent variable from several demographic and socioeconomic variables. For all the categorical variables, the lowest coded category was the reference. The level of statistical significance was set at a probability of P < 0.05 for all tests. The univariate analyses were screened to identify variables to be included in the multiple linear analyses, and all variables whose p-value approached significance at 10% underwent multiple regression analyses [39, 40]. However, the level of statistical significance for establishing an association was set at a probability of P < 0.05 for all tests.

## Results

### Participant demographics

The characteristics of the study’s participants are summarised in Table 1. A total of 663 youth and young adults participated in our study, of whom 650 provided data on their gender (44.9% were female). One in three (33.2%) surveyed young people were aged 13–17 years, while 66.2% were aged 18–24 years, with the average age of the study sample being 19.5 (95%CI: 19.3, 19.8) years. The mean age of educational attainment was 9.2 (95%CI: 8.9, 9.5) years, that is, lower secondary level, with 39.6%, 31.4%, 15.3% and 13.7% completing seven years or less (primary or less), 8–11 years (lower secondary), 12–13 years (upper secondary) and 14 years or more (post-secondary) of schooling respectively. Only a quarter of the surveyed participants (24.9%) lived in a nuclear family (i.e. with father and mother) while 24.6% lived in a single parent-headed family, 23% lived alone, 20.5% lived with relatives, and 7.1% lived with non-relatives. The proportion of youth and young adults identifying themselves as having disability was 7.5%.

**Table 1:** Association between the sexual behaviours and demographic and socio-economic characteristics.

Characteristic	Statistics	Ever had sexual intercourse		Used condom during the last sexual encounter		Had sexual intercourse in last 12 months		Mean number of sexual partner in last 12 months	
	N (%)	N (%)	AOR	N (%)	AOR	N (%)	AOR	Mean (SD)	Aβ
All	663 (100)	405 out of 657 (62.6)		218 out of 403 (54.1)		370 out of 401 (92.3)			
**Gender**									
Female	292 (44.9)	256 (64.7)	Ref	184 (48.4)	Ref	185 (97.3)	Ref	1.6 (1.0)	Ref
Male	358 (55.1)	349 (61.3)	0.72 (0.47, 1.13)	214 (58.4)	1.40 (0.87, 2.27)	210 (87.6)	**0.14 (0.04, 0.44)**	1.9 (1.1)	*0*.*31 (0*.*06*, *0*.*56)*
**Age**									
18–25 years	439(66.8)	425(80.5)	Ref	340 (54.4)	Ref	338 (93.2)	Ref	1.8 (1.1)	Ref
13–17 years	218 (33.2)	216 (26.9)	**0.22 (0.13, 0.37)**	58 (53.5)	0.99 (0.49, 1.97)	58 (87.9)	0.90 (0.28, 2.93)	1.5 (0.8)	-0.25 (-0.61, 0.11)
**Disability**									
No	607 (92.5)	594 (63.5)	Ref	377 (53.9)	Ref	375 (92.5)	Ref	1.8 (1.1)	Ref
Yes	49 (7.5)	47(48.9)	0.60 (0.27, 1.34)	22 (59.1)	0.95 (0.33, 2.74)	22 (86.4)	0.72 (0.13, 4.01)	1.6 (0.9)	-0.17 (-0.71, 0.38)
**Education**									
Post-secondary	90 (13.7)	85 (80.0)	Ref	69 (65.2)	Ref	68 (95.6)	Ref	1.6 (1.0)	Ref
Upper secondary	101(15.3)	97(67.0)	0.70 (0.32, 1.54)	64 (76.6)	2.11 (0.89, 5.03)	64 (90.6)	0.5 (0.11, 2.61)	1.6 (0.9)	-0.11 (-0.51, 0.29)
Lower secondary	207(31.4)	206 (72.8)	1.15 (0.54, 2.43)	147 (50.3)	*0*.*46 (0*.*23*, *0*.*90)*	147 (92.5)	0.73 (0.16, 3.24)	1.8 (1.1)	0.06(-0.29, 0.41)
Primary or less	261 (39.6)	255 (46.7)	***0*.*39 (0*.*18*, *0*.*85)***	120 (39.2)	*0*.*45 (0*.*21*, *0*.*94)*	119 (91.6)	0.49 (0.10, 2.41)	1.9 (1.2)	0.19 (-0.19, 0.57)
**Living arrangements (Live)**									
With mother and father	152 (24.9)	148 (48.0)	Ref	71 (67.6)	Ref	71 (91.6)	Ref	1.8 (1.1)	Ref
With mother only	123 (20.2)	120 (43.0)	0.83 (0.44, 1.56)	50 (72.0)	1.28 (0.54, 3.05)	50 (86.0)	0.31 (0.07, 1.30)	1.7 (1.0)	-0.09 (-0.53, 0.35)
With father only	27 (4.4)	26 (50.0)	1.43(0.50, 3.99)	13 (53.9)	0.60 (0.17, 2.15)	13 (84.6)	0.33(0.05, 2.39)	1.6 (1.2)	-0.19(-0.87, 0.49)
Alone	140 (23.0)	135 (85.2)	**2.76(1.36, 5.61)**	116 (52.6)	0.57(0.29, 1.15)	114 (96.5)	1.10(0.22, 5.41)	1.8 91.0)	-0.11(-0.45, 0.23)
With relatives	125 (20.5)	123 (62.6)	1.35(0.72, 2.53)	77 (49.4)	0.60(0.28, 1.25)	75 (93.3)	0.43(0.09, 1.97)	1.6 (1.0)	-0.08(-0.45, 0.29)
With non-relatives	43 (7.1)	42 (69.1)	1.73(0.68, 4.41)	28 (17.7)	**0.13(0.04, 0.44)**	29 (82.8)	0.18(0.03, 1.03)	2.4 (1.3)	0.49(-0.03, 1.01)
**Employment status**									
Paid employment	132 (20.3)	131(77.9)	Ref	102 (56.7)	Ref	101 (94.1)	Ref	1.6 (0.9)	Ref
Self-employed	165 (25.4)	159 (82.4)	1.37(0.69, 2.72)	133 (51.1)	0.70(0.37, 1.33)	132 (93.9)	1.11(0.29, 4.25)	1.7 (1.1)	0.20(-0.12, 0.52)
Still at school	190 (29.2)	186 (28.5)	**0.27(0.14, 0.50)**	52 (69.2)	0.87(0.38, 1.97)	52 (82.7)	*0*.*20(0*.*05*, *0*.*82)*	1.6 (0.9)	0.16(-0.26, 0.57)
Unemployed	163 (25.1)	159 (69.8)	1.19(0.62, 2.30)	108 (48.2)	0.71(0.36, 1.37)	108 (93.5)	0.64(0.18, 2.30)	2.0 (1.2)	0.39(0.05, 0.73)

Note: AOR = Adjusted odds ratios, Aβ = Adjusted Beta coefficients, **Bold** = p<0.001, **Bold and italic** = p<0.01, **Italic** = p<0.05.

### Sexual behaviours and source of information about sexual education

The study found that the majority of participants in the study area were sexually active, with 405 of the 647 respondents (62.6%; 95%CI: 58.8%, 66.3%) reporting having ever had sex. Bivariate analyses found that the proportion of participants who have ever had sex did not vary by gender, but significantly differed according to whether or not the participant had a disability, living arrangements, educational attainment, and employment status (Table 1). These results remain consistent after adjusting for socio-demographic and economic factors. In the adjusted model (data not shown) containing gender, age, whether or not the participant had a disability, educational attainment, living arrangements, and employment status (Pseudo R^2^ = 0.300), the odds of reporting ever having had sexual intercourse were lower among 13–17 year old participants (OR: 0.22; 95%CI: 0.13, 0.37, p<0.001), participants who never went beyond primary school level (OR: 0.39, 95%CI: 0.18, 0.85; p<0.05), and participants still at school (OR: 0.27, 95%CI: 0.14, 0.50; p<0.001) when compared with participants aged 18–24 years, with post-secondary education, and with full time paid employment respectively. However, the odds of reporting ever having had sexual intercourse were higher among children and young adults living alone (OR: 2.75; 95%CI: 1.35, 5.61; p<0.01) than those living in a nuclear family. The effect of disability became non-significant.

The mean age of sexual debut was 16 years (95%CI: 15.6, 16.4 years, range: 5–23 years), and there was a slight difference between males (15.8 years, 95%CI = 15.3, 16.4) and females (16.2 years, 95%CI: 15.7, 16.7). Sexual debut occurred at a very young age, with 6% being nine years or younger, 10.6% being 10–13 years, and 46.6% being 14 to 17 years, and 36.8% experiencing sexual debut at 18 years or older. Just over half of those who were sexually active (54.1%; 95%CI: 49.2, 58.9) had used condoms the last time they had sexual intercourse (48.4% for female vs 58.4% for male, p<0.05). Low levels of condom use the last time participants had sexual intercourse was associated with a low level of educational attainment and living with relatives, before and after adjusting for covariates. While the proportion of boys reporting using condoms the last time they had sexual intercourse was higher than that of girls (58.4% vs. 48.4%, p<0.05), this difference disappeared in the adjusted model.

Of those who reported being sexually active, 390 out of 405 (92.3%; 95%CI: 89.2, 94.5) reported having had sexual intercourse in the last 12 months preceding the survey, and the prevalence was significantly lower among boys than girls (87.6% vs. 97.3%, p<0.001), and among those still at school when compared to those in the workforce (82.7% vs. 94.0% p<0.05). Adjusting for covariates did not make any difference.

In the 12 months preceding the survey sexually active youth and young adults reported an average of 1.8 partners (95%CI: 1.7, 1.9; range 1–4) but the average number of sexual partners was significantly lower for females than for males (1.6 vs.1.9; p<0.05). Of the 390 participants who were sexually active in the 12 months preceding the survey, more than half (58.9%) had one sexual partner, while 19.2% had two sexual partners, 8.9% had three sexual partners, and 13% had four sexual partners. Among males, the number of sexual partners was closely associated with employment status, with low number of sexual partners associated with being unemployed.

While 28.9% of respondents did not know if their most recent sexual partner was much older or younger, 18.1% reported an age gap of 10 years or older, 4.5% reported 6–9 years, 35.2% reported 2–5 years and 13.4% reported one year or less.

The main sources of information about sexual education were school teachers (73.9%), parents and family members (40.9%), friends (26.2%), doctors or health professionals (26.1%), internet (25.3%), community outreach officers (25.0%), parent support groups (14.7%), books/magazines (8.6%), and television/films/videos/radio programs (3.8%).

### Sexual consent as a right and gender-based violence

Data on sexual consent as a right and gender-based violence are summarised in Tables 2, 3 and 4. More than three quarters (80.6%) of the participants who reported ever having sexual intercourse had willing (i.e. consensual) first sexual intercourse, although this proportion was significantly lower among girls than boys (69.8% vs. 90.2%, p<0.001). Low prevalence of willing first sexual intercourse was associated with younger age (OR = 0.48, 95%CI: 0.25, 0.90, p<0.05), having a disability (OR = 0.40, 95%CI: 0.16, 0.98, p<0.05), living with non-relatives (OR = 0.44, 95%CI: 0.16, 0.97, p<0.05), and being still at school (OR = 0.29, 95%CI: 0.12, 0.67, p<0.01). These results remained significant after adjusting for covariates, except for disability and the age of participants. Significant proportions of participants reported being persuaded to have sex through gifts, money or other favours (7.4%) (95%CI: 5.2, 10.5); tricked or deceived (5%) (95%CI: 3.3, 7.8); while 6.8% (95%CI: 4.8, 9.9) were forced to have sex and raped during their first sexual intercourse. The proportion of unwilling first sexual intercourse varied significantly by gender, with the proportion of sexually active women reporting being persuaded (13.2% vs. 2.4%, p<0.001), tricked (7.1% vs 2.9%, p<0.05) and forced or raped (9.9% vs 4.4%, p<0.05) at first sexual intercourse being significantly higher than that reported by sexually active men. However, apart from persuasion, these results became non-significant after adjusting for covariates.

**Table 2:** Association between the experience of the first of sexual intercourse and demographic and socio-economic characteristics (N = 392).

Characteristic	Willing participant without fear or gifts		Persuaded through gifts, money or other favours		Tricked/deceived		Forced or raped	
All	UOR	AOR	UOR	AOR	UOR	AOR	UOR	AOR
**Gender**								
Male	Ref	Ref	Ref	Ref	Ref	Ref	Ref	Ref
Female	**0.25(0.14, 0.44)**	**0.31(0.17, 0.57)**	**6.08(2.27, 16.28)**	***4*.*66(1*.*59*. *13*.*64)***	2.55(0.96, 6.85)	*3*.*12(1*.*08*, *8*.*98)*	*2*.*39(1*.*05*, *5*.*46)*	1.69(0.67, 4.28)
**Age**								
18–25 years	Ref	Ref	Ref	Ref	Ref	Ref	Ref	Ref
13–17 years	*0*.*48 (0*.*25*, *0*.*90)*	0.62(0.27, 1.38)	***3*.*14(1*.*34*, *7*.*36)***	2.41(0.79, 7.41)	0.30(0.04, 2.28)	0.15(0.02, 1.38)	2.34(0.94, 5.87)	2.59(0.82, 8.20)
**Disability**								
No	Ref	Ref	Ref	Ref	Ref	Ref	Ref	Ref
Yes	*0*.*40(0*.*16*, *0*.*98)*	0.72(0.22, 2.37)	0.57(0.07, 4.43)	0.43(0.05, 4.03)	***4*.*86(1*.*47*, *16*.*04)***	4.26(0.91, 19.99)	2.25(0.62, 8.14)	0.62(0.07, 5.33)
**Education**								
Post-secondary	Ref	Ref	Ref	Ref	Ref	Ref	Ref	Ref
Upper secondary	0.61(0.26, 1.47)	0.72(0.27, 1.92)	2.86(0.53, 15.31)	1.86(0.30, 11.68)	2.72(0.67, 11.04)	4.69(0.99, 22.18)	0.52(0.12, 2.17)	0.29 (0.06, 1.44)
Lower secondary	0.96(0.44, 2.09)	1.11(0.45, 2.76)	1.87(0.39, 9.05)	1.49(0.26, 8.57)	0.75(0.17, 3.24)	1.17(0.22, 6.21)	0.90(0.32, 2.52)	0.52(0.15, 1.76)
Primary or less	0.74(0.34, 1.61)	1.32(0.49, 3.54)	4.35(0.96, 19.77)	1.86(0.31, 11.11)	0.94(0.22, 4.05)	1.93(0.35, 10.77)	0.54(0.17, 1.75)	*0*.*19(0*.*04*, *0*.*89)*
**Living structure (Live)**								
With mother and father	Ref	Ref	Ref	Ref	Ref	Ref	Ref	Ref
With mother only	0.54(0.22, 1.32)	0.74(0.28, 2.00)	2.89(0.80, 10.49)	1.77(0.42, 7.51)	0.73 (0.13, 4.17)	0.41(0.06, 2.92)	1.53 (0.36, 6.47)	1.74(0.38, 7.89)
With father only	1.14(0.22, 5.81)	0.91(0.17, 4.96)	1.38(0.14, 13.29)	2.01(0.19, 21.36)	1.00(0.24, 3.75)	1.01(0.28, 4.87)	1.38(0.14, 13.39)	1.48(0.14, 15.63)
Alone	1.37(0.60, 3.12)	1.05(0.41, 2.66)	0.76 (0.20, 2.92)	0.99(0.21, 4.54)	1.08(0.30, 3.83)	1.28(0.32, 4.22)	0.45(0.10, 2.05)	0.41(0.07, 2.52)
With relatives	0.59(0.26, 1.32)	0.54(0.22, 1.34)	1.21(0.31, 4.72)	1.07(0.25, 4.64)	1.21(0.31, 4.72)	0.91(0.20, 4.22)	2.32(0.68, 7.91)	3.23(0.85, 12.59)
With non-relatives	*0*.*44(0*.*16*, *0*.*97)*	*0*.*31(0*.*10*, *0*.*92)*	2.75(0.64, 11.87)	3.19(0.61, 16.53)	1.27(0.22, 7.36)	1.25(0.18, 8.90)	1.98(0.41, 9.48)	3.86(0.68, 22.04)
**Employment status**								
Paid employment	Ref	Ref	Ref	Ref	Ref	Ref	Ref	Ref
Self-employed	0.72 (0.34, 1.55)	0.73(0.30, 1.75)	2.36(0.62, 8.94)	2.46(0.56, 10.78)	0.94(0.25, 3.60)	0.79(0.17, 3.58)	1.06(0.33, 3.44)	1.33(0.34, 5.25)
Still at school	***0*.*29(0*.*12*, *0*.*67)***	*0*.*36(0*.*14*, *0*.*95)*	*4*.*42(1*.*06*, *18*.*50)*	3.28(0.66, 16.23)	2.67(0.68, 10.43)	3.37(0.69, 16.57)	2.11(0.58, 7.68)	1.77(0.41, 7.60)
Unemployed	*0*.*44(0*.*21*, *0*.*93)*	0.60(0.26, 1.41)	3.23(0.86, 12.10)	1.92(0.44, 8.35)	1.38(0.38, 5.05)	1.51(0.36, 6.43)	1.90(0.63, 5.76)	1.50(0.42, 5.42)

**Bold** = p<0.001, **Bold and italic** = p<0.01, **Italic** = p<0.05.

**Table 3:** Association between sexual consent as a right and gender-based violence and demographic and socio-economic characteristics.

Characteristic	Would not be able to stop someone trying to have sexual intercourse with me without consent (N = 654)		A boy and a girl who are friends should have sex before they become engaged to see whether they are suited to each other		It is sometimes justifiable for a boyfriend/husband to hit his girlfriend/wife, as long as they love each other		It is not important that you should fall in love with someone first before having sexual intercourse with them	
All	UOR	AOR	UOR	UOR	UOR	AOR	UOR	AOR
**Gender**								
Male	**Ref**	**Ref**	**Ref**	**Ref**	**Ref**	**Ref**	**Ref**	**Ref**
Female	1.11(0.62, 2.01)	1.15(0.61, 2.18)	*0*.*69(0*.*50*, *0*.*97)*	*0*.*71(0*.*51*, *0*.*98)*	*0*.*71(0*.*51*, *0*.*98)*	0.92(0.60, 1.41)	*0*.*71(0*.*51*, *0*.*98)*	0.77(0.53, 1.11)
**Age**								
18–25 years	**Ref**	**Ref**	**Ref**	**Ref**	**Ref**	**Ref**	**Ref**	**Ref**
13–17 years	*2*.*03(1*.*13*, *3*.*66)*	1.69(0.73, 3.89)	*0*.*63(0*.*43*, *0*.*92)*	1.03(0.71, 1.48)	1.03(0.71, 1.48)	1.14(0.67, 1.94)	1.03(0.71, 1.48)	1.00(0.61, 1.63)
**Disability**								
No	**Ref**	**Ref**	**Ref**	**Ref**	**Ref**	**Ref**	**Ref**	**Ref**
Yes	0.54(0.13, 2.24)	0.45(0.10, 2.00)	1.09(0.56, 2.10)	1.13(0.59, 2.19)	1.13(0.59, 2.19)	0.89(0.39, 2.04)	1.13(0.59, 2.19)	1.17(0.56, 2.42)
**Education**								
Post-secondary	**Ref**	**Ref**	**Ref**	**Ref**	**Ref**	**Ref**	**Ref**	**Ref**
Upper secondary	0.37(0.09, 1.48)	0.46(0.11, 2.01)	1.26(0.69, 2.31)	0.81(0.45, 1.46)	0.81(0.45, 1.46)	*3*.*21(1*,*09*, *9*.*42)*	0.81(0.45, 1.46)	0.85(0.45, 1.61)
Lower secondary	0.86(0.33, 2.21)	0.97(0.32, 2.94)	*1*.*67(1*.*00*, *2*.*82)*	1.35(0.81, 2.26)	1.35(0.81, 2.26)	**6.82(2.54, 18.27)**	1.35(0.81, 2.26)	1.53(0.86, 2.73)
Primary or less	1.26(0.52, 3.02)	1.17(0.38, 3.56)	1.31(0.67, 1.90)	1.38(0.83, 2.29)	1.38 (0.83, 2.29)	**7.80(2.86, 21.30)**	1.38(0.83, 2.29)	1.39(0.76, 2.54)
**Living structure (Live)**								
With mother and father	**Ref**	**Ref**	**Ref**	**Ref**	**Ref**	**Ref**	**Ref**	**Ref**
With mother only	1.78(0.69, 4.57)	1.77(0.66, 4.70)	0.83(0.48, 1.42)	0.82(0.46, 1,45)	1.08(0.60, 1.97)	0.87(0.45, 1.65)	1.37(0.81, 2.33)	1.49(0.84, 2.61)
With father only	1.41(0.28, 7.03)	1.48(0.29, 7.59)	1.23(0.51, 3.01)	1.16(0.46, 1.45)	1.29(0.49, 3.39)	1.15(0.42, 3.17)	1.51(0.61, 3.75)	1.57(0.62, 3,98)
Alone	1.53(0.60, 3.91)	2.78(0.92, 8.46)	1.60(0.97, 2.64)	1.50(0.85, 2.65)	0.93(0.52, 1.65)	0.93(0.48, 1.82)	*1*.*87(1*.*14*, *3*.*09)*	*2*.*05(1*.*17*, *3*.*60)*
With relatives	1.37(0.51, 3.65)	1.48(0.51, 4.33)	1.45(0.86, 2.43)	1.52(0,87, 2.67)	1.48(0.84, 2.62)	1.29(0.69, 2.42)	1.10(0.65, 1.86)	1.30(0.74, 2.29)
With non-relatives	*3*.*53(1*.*20*, *10*.*38)*	***4*.*67(1*.*32*, *16*.*56)***	1.57(0.76, 3.24)	1.46(0.64, 3.30)	1.39(0.63, 3.07)	0.76(0.31, 1.87)	***2*.*08(1*.*00*, *4*.*34)***	2.07(0.91, 4.71)
**Employment status**								
Paid employment	**Ref**	**Ref**	**Ref**	**Ref**	**Ref**	**Ref**	**Ref**	**Ref**
Self-employed	0.43(0.14, 1.31)	0.40(0.12, 1.41)	1.26(0.78, 2.03)	1.21(0.71, 2.08)	1.48(0.83, 2.63)	1.26(0.66, 2.37)	1.37(0.84, 2.21)	1.42(0.82, 2.44)
Still at school	1.64(0.72, 3.73)	1.77(0.65, 4.81)	0.85(0.52, 1.37)	1.21(0.69, 2.13)	1.22(0.68, 2.18)	0.92(0.46, 1,82)	1.21(0.75, 1.96)	1.50(0.84, 2.65)
Unemployed	1.28(0.54, 3.06)	1.22(0.47, 3.22)	1.38(0.85, 2.25)	1.66(0.95, 2.90)	***2*.*62(1*.*49*, *4*.*59)***	1.85(0.99, 3.47)	1.37(0.84, 2.22)	1.25(0.72, 2.17)

**Bold** = p<0.001, **Bold and italic** = p<0.01, **Italic** = p<0.05.

**Table 4:** Association between sexual and reproduction health needs and rights and demographic and socio-economic characteristics.

	All	Female	Male	P-value	13–17 years	18–25 years	P-value
Sexual and reproduction health right and needs							
If someone tried to have sexual intercourse with you or touch you sexually and you did not want them to, would you be able to safely stop them? **% answering “definitely not” N (%)**	49 out of 654 (7.4%)	23 out of 291 (7.9%)	25 out of 350(7.1%)	0.716	24 out of 216 (11.1%)	25 out of 432 (5.8%)	<0.01
*Attitudes and beliefs*							
It is common for young females to be forced to have sexual intercourse against their will by a stranger, a relative or an older person	447 out of 610(73.3%)	205 out of 271(75.7%)	237 out of 329(72.0%)	0.318	126 out of 182 (69.2%)	317 out of 423(74.9%)	0.146
A girl can safely suggests to her boyfriend that he uses a condom without fear and hesitation	433 out of 561(77.2%)	198 out of 254(78.0%)	231 out of 299(77.3%)	0.845	115 out of 152(75.7%)	315 out of 405(77.8%)	0.595
You can safely refuse to have sex with someone who is not prepared to use a condom no matter what, without fear	435 out of 549(79.2%)	203 out of 253(80.2%)	226 out of 289(78.2%)	0.560	125 out of 148(84.5%)	308 out of 399(77.2%)	0.063
A boy will not respect a girl who agrees to have sex with him but she insists on him using a condom	263 out of 542(48.5%)	126 out of 242(52.1%)	133 out of 294(45.2%)	0.115	75 out of 150(50.0%)	185 out of 389(47.6%)	0.611
It is sometimes okay for a boy to force a girl to have sex if he loves her, it does not matter whether she has some feeling for him	202 out of 557(34.3%)	92 out of 269(34.2%)	107 out of 309(34.6%)	0.914	52 out of 160(32.5%)	149 out of 422(35.3%)	0.525
It is sometimes justifiable for a boy/husband to hit his girlfriend/wife, as long as they love each other	153 out of 582 (26.3%)	68 out of 261(26.1%)	83 out of 313(26.5%)	0.900	54 out of 167(32.3%)	98 out of 410(23.9%)	<0.05
If a sexual partner (not married) becomes accidently pregnant, both the girlfriend and the boyfriend would never contemplate having an abortion	272 out of 523(52.0%)	121 out of 241(50.2%)	147 out of 276(53.3%)	0.488	68 out of 133(51.1%)	202 out of 387(52.2%)	0.831
It is mainly the woman's responsibility to ensure that contraception is used regularly to prevent unwanted pregnancy and/or diseases	352 out of 561(62.8%)	173 out of 255(67.8%)	175 out of 298(58.7%)	<0.05	84 out of 147(57.1%)	266 out of 409(65.0%)	0.089
It is not important that you should fall in love with someone first before having sex with them	269 out of 571(47.1%)	111 out of 262(42.4%)	153 out of 300(51.0%)	<0.05	75 out of 158(47.8%)	191 out of 408(46.8%)	0.889
*Access to contraceptive services*, *family planning*, *and prevention of STDs*							
If you are planning birth control you know of a place where you can get condoms, pills, IUD, or implant [P	382 out of 499(76.6%)	189 out of 236(80.1%)	187 out of 254(73.6%)	0.091	76 out of 121(62.8%)	302 out of 374(80.8%)	<0.001
In the last 12 months you’ve visited a health facility to get information on family planning and STDs	285 out of 587(48.6%)	141 out of 267(52.8%)	141 out of 311(45.3%)	0.073	60 out of 161(37.3%)	223 out of 422(52.8%)	<0.01
If you have a question about abortion, you have somewhere or someone nearby you can go to for help	311 out of 467(66.6%)	151 out of 222(68.0%)	154 out of 23(65.0%)	0.491	69 out of 111(62.2%)	239 out of 352(67.9%)	0.264
You can make decisions about whether or not, and when, to have children without fear [S]	411 out of 517(79.5%)	185 out of 238(77.7%)	220/272(80.9%)	0.380	91 out of 129(70.5%)	316 out of 384(82.3%)	<0.01
*HIV Testing counselling*, *disclosure and support*							
If you were to undertake an HIV you are confident that you will be given pre& post testing counselling	418 out of 519(80.5%)	190 out of /235(80.6%)	223 out of /276(80.8%)	0.988	94 out of 130(72.3%)	320 out of 385(83.1%)	<0.01
You would be able to safely disclose the result of your HIV test to your partner (if he or she was not there at the time of the test) if you wanted to, without fear	377 out of 511(73.8%)	178 out of 237(75.1%)	193 out of 267(72.3%)	0.473	99 out of 139(71.2%)	276 out of 370(74.6%)	0.442
In case of a positive HIV diagnosis, there are HIV treatment, care and support services in your community	380 out of 506(75.1%)	193 out of 238(81.1%)	182 out of 260(70.0%)	<0.01	94 out of 124(75.8%)	284 out of 379(74.9%)	0.845

Reasons for the sexual intercourse in the last 12 months preceding the survey followed a similar pattern, with the proportion of participants reporting willing sexual intercourse estimated at 89.1% (female: 82.0% vs male: 95.7%, p<0.001), while the proportion of unwilling sexual encounter was estimated at 5.7% (female: 10.5% vs male: 1.6%, p<0.001) for persuasion, 2.5% (female: 3.5% vs male: 1.1%, p = 0.119) for tricked or deceived, and 2.7% (female: 4.1% vs male: 1.6%, p<0.05) for forced and raped.

One in 13 participants (7.4%) indicated that, if someone tried to have sexual intercourse with them or touch them sexually without their consent, they would definitely not be able to prevent it happening, and this was more significantly so among 13–17 year old participants than their older counterparts (11.1% vs 5.8%, p<0.01). Although 77.2% of young people indicated that a girl can confidently suggest to her boyfriend to use a condom without fear or hesitation, some 48.5% of respondents indicated that a boy will not respect a girl who agrees to have sex with him if she insists on him using a condom. One third of participants (34.3%) affirmed that it was alright for a boy to force a girl to have sex if he had feelings for her, even if she didn’t have the same feelings for him, while three quarters of respondents (73.3%) also believed it was common for strangers and relatives to force young females to have sexual intercourse with them without consent. Some 26.3% of participants indicated that it was sometimes justifiable for a boy to hit his girlfriend, as long as they loved each other. These held beliefs and attitudes did not vary by gender or age, hence highlighting the tolerance and endorsement of culturally-mediated gender-based sexual violence. Approximately half (52.0%) of the study’s participants affirmed that they would never contemplate having an abortion if a sexual partner (not married) became accidently pregnant; while 62.8% (female: 67.8% vs male: 58.7%, p<0.05) agreed that it was mainly the woman's responsibility to ensure that contraception was used regularly to prevent unwanted pregnancy and/or diseases.

### Access to contraceptive services, family planning, and prevention of STDs

**Fig 1:**
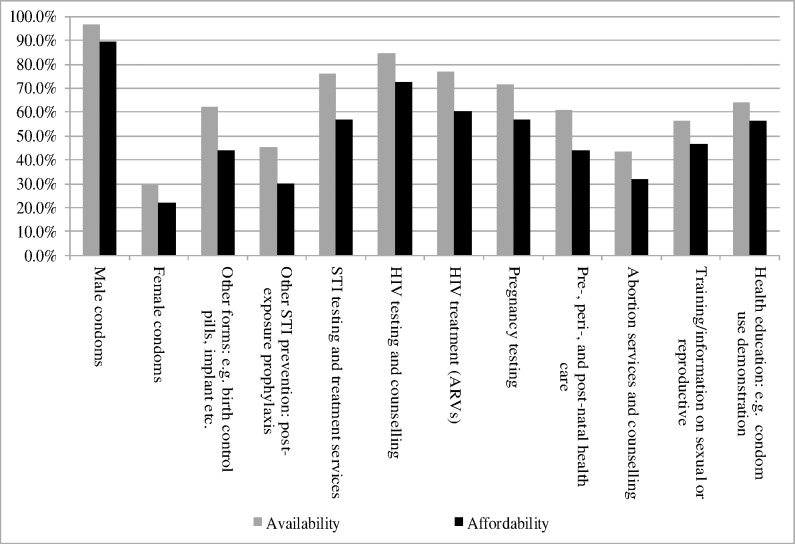
Access to and affordability of birth control and STDs prevention products and services.

Data on access to contraceptive services, family planning, and prevention of STDs are summarised in Table 5 and Fig 1. The majority of participants (76.6%) knew where and how to access condoms, pills, intrauterine devices, or birth control implants for planning birth control purposes, but the proportion was significantly lower among 13–17 year old participants than their older counterparts (62.8% vs 80.8%, p<0.001). Just half (48.6%) of participants had visited a health facility to get information on family planning and STDs in the last 12 months preceding the survey, with younger participants less likely than their older counterparts to visit a health facility (37.3% vs 52.8%, p<0.01). Two in three (66.6%) study participants knew of somewhere (or someone) nearby where they could go to get help if they needed information concerning abortion, or to obtain an abortion. More than three quarters (79.5%) affirmed that they could make decisions about whether or not, and when, to have children without fear. Data in Fig 1 indicates that female condoms, post-exposure prophylaxis, abortion services and counselling were not readily available and accessible. In addition, those services that were highly available were not necessarily affordable, especially post-exposure prophylaxis, STIs testing and counselling, HIV treatment, birth control pills and implants, pre-, peri-, and post-natal health care, and pregnancy testing.

**Table 5:** Association between access to contraceptive services and family planning, prevention of STDs, and demographic and socio-economic characteristics.

Characteristic	It is mainly the woman's responsibility to ensure that contraception is used regularly to prevent unwanted pregnancy and/or diseases		If a situation arose where you have to use a condom, you know where and how to access it easily		In the last 12 months you’ve visited a health facility to get information on family planning and STDs		If you are planning birth control you know of a place where you can get condoms, pills, IUD, or implant	
All	UOR	AOR	UOR	AOR	UOR	AOR	UOR	AOR
**Gender**								
Male	**Ref**	**Ref**	**Ref**	**Ref**	**Ref**	**Ref**	**Ref**	**Ref**
Female	*1*.*48(1*.*05*, *2*.*10)*	1.41(0.95, 2.09)	*0*.*56(0*.*37*, *0*.*88)*	*0*.*56(0*.*34*, *0*.*93)*	1.35(0.97, 1.87)	1.27(0.87, 1.84)	1.92(0.94, 2.20)	*1*.*68(1*.*03*, *2*.*75)*
**Age**								
18–25 years	**Ref**	**Ref**	**Ref**	**Ref**	**Ref**	**Ref**	**Ref**	**Ref**
13–17 years	0.72(0.49, 1.05)	0.82 (0.50, 1.37)	**0.40(0.26, 0.63)**	0.57(0.31, 1.04)	**0.53(0.37, 0.77)**	*0*.*54(0*.*33*, *0*.*89)*	**0.40(0.26, 0.63)**	*0*.*53(0*.*29*, *0*.*98)*
**Disability**								
No	**Ref**	**Ref**	**Ref**	**Ref**	**Ref**	**Ref**	**Ref**	**Ref**
Yes	0.89(0.44, 1.80)	1.05 (0.47, 2.35)	1.22(0.49, 3.01)	2.56 (0.82, 7.92)	***2*.*54(1*.*29*, *4*.*99)***	***2*.*83(1*.*36*, *5*.*92)***	0.51(0.25, 1.04)	0.54(0.24, 1.19)
**Education**								
Post-secondary	**Ref**	**Ref**	**Ref**	**Ref**	**Ref**	**Ref**	**Ref**	**Ref**
Upper secondary	*2*.*00(1*.*07*, *3*.*75)*	*2*.*30(1*.*17*, *4*.*52)*	0.45(0.16, 1.24)	0.40(0.13, 1.24)	0.99(0.56, 1.77)	1.08(0.57, 2.01)	0.97(0.39, 2.42)	1.03(0.40, 2.65)
Lower secondary	1.38(0.82, 2.34)	1.73(0.96, 3.14)	***0*.*32(0*.*12*, *0*.*79)***	*0*.*35(0*.*12*, *0*.*99)*	1.09(0.66, 1.81)	1.17(0.66, 2.08)	*0*.*42(0*.*20*, *0*.*88)*	0.60(0.27, 1.35)
Primary or less	1.14(0.68, 1.92)	1.15(0.62, 2.16)	**0.17(0.07, 0.42)**	***0*.*17(0*.*06*, *0*.*48)***	0.62(0.38, 1.03)	0.71(0.39, 1.30)	**0.23(0.11, 0.49)**	***0*.*32(0*.*14*, *0*.*72)***
**Living structure (Live)**								
With mother & father	**Ref**	**Ref**	**Ref**	**Ref**	**Ref**	**Ref**	**Ref**	**Ref**
With mother only	0.94(0.54, 1.63)	0.90(0.49, 1.63)	0.77(0.40, 1.47)	0.86(0.42, 1.78)	1.16(0.69, 1.95)	1.23(0.70, 2.15)	0.80(0.42, 1.50)	1.12(0.55, 2.30)
With father only	0.61(0.24, 1.51)	0.62(0.24, 1.60)	1.29(0.35, 4.81)	1.35(0.35, 5.20)	0.94(0.39, 2.29)	1.11(0.44, 2.78)	0.88(0.26, 2.96)	1.23(0.33, 4.55)
Alone	1.11(0.66, 1.86)	1.08(0.61, 1.94)	*2*.*32(1*.*10*, *4*.*91)*	1.80(0.77, 4.23)	0.82(0.50, 1.33)	0.66(0.38, 1.16)	*1*.*95(1*.*00*, *3*.*83)*	2.00(0.92, 4.32)
With relatives	1.32(0.76, 2.29)	1.26(0.69, 2.29)	0.69(0.37, 1.29)	0.88(0.43, 1.80)	0.87(0.52, 1.45)	0.79(0.45, 1.38)	0.80(0.43, 1.48)	1.00(0.50, 2.01)
With non-relatives	0.89(0.42, 1.90)	0.98(0.41, 2.30)	0.91(0.37, 2.24)	1.19(0.43, 3.30)	0.62(0.30, 1.28)	0.60(0.26, 1.38)	0.64(0.27, 1.52)	0.97(0.36, 2.65)
**Employment status**								
Paid employment	**Ref**	**Ref**	**Ref**	**Ref**	**Ref**	**Ref**	**Ref**	**Ref**
Self-employed	0.93(0.57, 1.52)	1.08(0.61, 1.88)	*1*.*92(1*.*01*, *3*.*65)*	2.05(0.94, 4.44)	1.47(0.92, 2.36)	1.70(0.99, 2.94)	1.61(0.88, 2.94)	1.78(0.88, 3.59)
Still at school	0.81(0.49, 1.33)	0.88(0.49, 1.60)	1.19(0.65, 2.19)	1.75(0.81, 3.77)	1.10(0.68, 1.77)	1.32(0.74, 2.35)	1.30(0.72, 2.36)	*2*.*20(1*.*04*, *4*.*64)*
Unemployed	1.29(0.77, 2.16)	1.45(0.81, 2.62)	0.82(0.46, 1.45)	0.98(0.49, 1.96)	1.26(0.78, 2.03)	1.44(0.82, 2.51)	0.94(0.53, 1.68)	1.39(0.70, 2.74)

**Bold** = p<0.001, **Bold and italic** = p<0.01, **Italic** = p<0.05.

### HIV testing counselling, disclosure and support

Data on HIV testing counselling, disclosure and support are summarised in Table 6. The majority of the study participants (80.5%) affirmed that they would be given pre- and post-testing counselling if they were to undertake an HIV test, and 73.8% affirmed that they would be able to safely disclose the result of their HIV test to their partner without fear, either if the partner was present at the time of the test, or if the partner was not with them at the test but was informed later. Three quarters of participants (75.1%) indicated that there were HIV treatment, care and support services in their community to serve those who become HIV+.

**Table 6:** Association between voluntary HIV testing, disclosure, and care and support and demographic and socio-economic characteristics.

Characteristic	If you have a question about STDs/HIV/AIDS, you have somewhere/someone nearby you can go to for help		You can make decisions about whether or not, and when, to have children without fear		If you were to undertake an HIV you are confident that you will be given pre& post testing counselling		In case of a positive HIV diagnosis, there are HIV treatment, care and support services in your community	
All	UOR	AOR	UOR	AOR	UOR	AOR	UOR	AOR
**Gender**								
Male	**Ref**	**Ref**	**Ref**	**Ref**	**Ref**	**Ref**	**Ref**	**Ref**
Female	1.40(0.91, 2.18)	*1*.*72(1*.*04*, *2*.*85)*	0.83(0.54, 1.27)	0.83(0.51, 1.36)	1.00(0.65, 1.56)	0.83(0.51, 1.37)	***1*.*84(1*.*21*, *2*.*79)***	*1*.*75(1*.*09*, *2*.*80)*
**Age**								
18–25 years	**Ref**	**Ref**	**Ref**	**Ref**	**Ref**	**Ref**	**Ref**	**Ref**
13–17 years	***0*.*55(0*.*35*, *0*.*87)***	0.81(0.44, 1.49)	*0*.*52(0*.*33*, *0*.*82)*	0.68(0.37, 1.25)	***0*.*53(0*.*33*, *0*.*85)***	0.73(0.38, 1.38)	1.05(0.65, 1.68)	1.35(0.71, 2.57)
**Disability**								
No	**Ref**	**Ref**	**Ref**	**Ref**	**Ref**	**Ref**	**Ref**	**Ref**
Yes	0.51(0.25, 1.05)	0.66 (0.30, 1.49)	0.75(0.34, 1.66)	0.97(0.40, 2.35)	0.84(0.37, 1.91)	0.77(0.32, 1.86)	1.05(0.46, 2.39)	1.03(0.43, 2.49)
**Education**								
Post-secondary	**Ref**	**Ref**	**Ref**	**Ref**	**Ref**	**Ref**	**Ref**	**Ref**
Upper secondary	1.12(0.44, 2.85)	1.28(0.48, 3.38)	1.11(0.47, 2.62)	0.99(0.40, 2.45)	1.00(0.37, 2.72)	0.78(0.27, 2.26)	1.24(0.57, 2.70)	1.34(0.59, 3.05)
Lower secondary	*0*.*42(0*.*19*, *0*.*87)*	0.61(0.27, 1.38)	0.67(0.33, 1.37)	0.75(0.34, 1.66)	0.47(0.21, 1.07)	*0*.*39(0*.*16*, *0*.*98)*	0.79(0.41, 1.52)	0.78(0.38, 1.60)
Primary or less	*0*.*42(0*.*20*, *0*.*88)*	0.54(0.23, 1.28)	***0*.*40(0*.*20*, *0*.*80)***	*0*.*41(0*.*18*, *0*.*92)*	**0.25(0.11, 0.56)**	**0.19(0.08, 0.48)**	***0*.*43(0*.*23*, *0*.*82)***	***0*.*36(0*.*17*, *0*.*76)***
**Living structure (Live…)**								
With mother and father	**Ref**	**Ref**	**Ref**	**Ref**	**Ref**	**Ref**	**Ref**	**Ref**
With mother only	0.62(0.33, 1.15)	0.75(0.38, 1.48)	0.72(0.37, 1.39)	0.79(0.39, 1.61)	1.23(0.62, 2.45)	1.68(0.79, 3.59)	1.06(0.56, 2.00)	1.02(0.50, 2.05)
With father only	1.34(0.36, 4.98)	1.71(0.44, 6.65)	1.01(0.31, 3.31)	1.17(0.35, 3.97)	0.82(0.27, 2.46)	0.88(0.27, 2.82)	1.79(0.48, 6.60)	2.39(0.59, 9.67)
Alone	***3*.*31(1*.*48*, *7*.*40)***	***3*.*18(1*.*31*, *7*.*70)***	1.19(0.62, 2.30)	1.08(0.51, 2.28)	1.03(0.55, 1.94)	0.98(0.47, 2.04)	0.84(0.47, 1.49)	1.02(0.52, 1.99)
With relatives	0.90(0.47, 1.72)	0.94(0.46, 1.92)	0.94(0.48, 1.82)	1.15(0.55, 3.28)	1.25(0.63, 2.48)	1.58(0.74, 3.34)	1.62(0.83, 3.16)	1.86(0.89, 3.87)
With non-relatives	0.54(0.22, 1.28)	0.54(0.20, 1.44)	1.18(0.44, 3.18)	1.61(0.55, 4.74)	0.89(0.34, 2.32)	2.16(0.68, 6.83)	1.62(0.40, 2.44)	1.55(0.54, 4.44)
**Employment status**								
Paid employment	**Ref**	**Ref**	**Ref**	**Ref**	**Ref**	**Ref**	**Ref**	**Ref**
Self-employed	1.26(0.66, 2.43)	1.41(0.66, 3.02)	0.81(0.42, 1.56)	0.84(0.40, 1.73)	1.24(0.68, 2.27)	1.58(0.80, 3.14)	1.00(0.58, 1.74)	1.49(0.79, 2.81)
Still at school	0.86(0.46, 1.63)	1.25(0.58, 2.70)	0.69(0.36, 1.34)	0.93(0.43, 2.02)	1.28(0.69, 2.36)	1.91(0.89, 4.10)	1.49(0.82, 2.71)	1.77(0.86, 3.65)
Unemployed	0.59(0.32, 1.10)	0.79(0.39, 1.61)	*0*.*51(0*.*27*, *0*.*97)*	0.60(0.29, 1.24)	1.15(0.61, 2.16)	1.77(0.85, 3.69)	1.32(0.73, 2.40)	1.86(0.94, 3.68)

**Bold** = p<0.001, **Bold and italic** = p<0.01, **Italic** = p<0.05

When asked which issues they would prioritize if given an opportunity to attend a meeting, HIV prevention topped the list, ahead of sexual and reproductive health needs, and followed by stopping gender-based violence, gender equality, and sexual and reproductive rights (Table 7). Three quarters (76.3%) of interviewed young people indicated knowing their health responsibilities, especially the responsibility to seek medical care, to follow treatment instructions, and to provide information. However, young people’s knowledge of Ugandan health policies was very poor.

**Table 7:** SHR priorities, health responsibilities, and health policy knowledge by gender and age.

	All	Female	Male	13–17 years	18–25 years
**SHR youth believe should be a priority (N = 657)**					
HIV prevention	81.7	80.6	82.3	80.6	82.3
Sexual and reproductive health needs (a)	61.4	61.5	61.4	*55*.*9*	*64*.*2*
Stopping gender-based violence	59.9	60.8	59.4	54.5	62.2
Gender equality	59.3	62.5	56.6	***50*.*7***	***63*.*0***
Sexual and reproductive rights (b)	54.4	55.6	53.5	*47*.*4*	*57*.*8*
Involvement of voiceless in decision-making processes	53.4	52.1	54.4	***44*.*6***	***57*.*5***
Integration of HIV services with Sexual and reproductive health services	48.2	49.3	47.0	**36.2**	**53.7**
Preventing traditional harmful practices such as FGM	43.3	42.4	43.9	*36*.*6*	*46*.*1*
Do you know your health responsibilities? (N = 651)	76.3	75.6	77.6	***69*.*6***	***79*.*6***
Seeking Medical care	79.9	79.8	80.7	78.1	80.6
Responsibility to follow treatment instructions	70.0	70.8	70.1	70.3	69.7
Responsibility to provide information e.g. name, age, illness history	60.6	58.1	62.8	60.4	60.3
Responsibility to respect others	54.0	53.6	55.6	58.3	52.1
Responsibility if you refuse treatment	47.6	48.3	47.4	46.4	47.9
Do you know any health Policies of this country? (N = 638)	24.0	24.7	24.0	19.4	26.3
Same sex relationship	16.4	17.9	15.4	*10*.*8*	*18*.*8*
Reproductive Health Policy	13.6	15.1	12.9	12.1	14.1
Adolescence sexual and reproductive Health Policy	11.4	11.5	11.4	8.3	12.9
National health Policy II	11.4	11.5	11.8	7.6	12.9
Health Sector Strategic plan (2010–2015)	11.2	14.2	8.8	10.2	11.5
Patients’ charter.	7.2	7.3	7.4	5.1	8.2
Uganda Health Management Committee handbook	6.8	7.8	6.3	8.9	5.6

(a) includes postponing parenthood, preventing unintended pregnancy, and the provision of essential sexual and reproductive health information and services

(b) the right to sexual a safe relationship without fear of infection or unwanted pregnancy, and a relationship free from coercion or violence.

**Bold** = p<0.001, **Bold and italic** = p<0.01, **Italic** = p<0.05

## Discussion

The study was the first effort to assess the sexual health needs and rights of young people in slum areas of Kampala using a large sample size. The use of a systematic random sampling method provided the opportunity to obtain a sample that is highly representative of the target population. The large sample size and the use of systemic random sampling make conclusions from our data valid. However, the study’s sample is limited to young people in slum areas of Kampala and our findings cannot be generalised among young people in Uganda. Our study was cross-sectional, hence limited to investigating the association between the independent and dependent variables, controlling for confounding factors. Causation cannot therefore be implied. Some of the data on sexual health needs and rights were collected retrospectively, hence it is possible that our data may have been subject to the recall bias. Notwithstanding these limitations, findings from this study have a wide range of policy implications.

Our study findings show that almost 81% of young people aged 18–24 years and 27% aged 13–17 years were sexually active. However, in both age groups condom use was only 54%. The strongest variable of condom use in sexual intercourse was its use in the last sexual encounter preceding the study. This is important because consistent and correct condom use prevents sexually transmitted diseases and early and unwanted pregnancies. Similar findings have been reported in youth studies, implying that sexually active youths who consistently use condoms protect themselves from sexually transmitted diseases and unwanted pregnancies [41–43].

Our data suggests that almost 77% of the sexually active respondents in upper secondary schools used condoms compared to 50% and 39% in lower secondary and primary schools. This implies that young people in upper secondary school gain critical knowledge which influences their sexual decisions. This is consistent with studies that found that sexuality education among the youth reduces risky behaviour, unwanted pregnancy and sexually transmitted diseases [44, 45]. However, it is possible that sexually active young people in lower secondary and primary schools have limited access to sex education and negotiation power over condom use, especially if their sexual partners are older. Lack of in-depth sex education and negotiation skills in lower secondary and primary schools exposes younger age groups at risk. Evidence suggests that sex education is most effective when delivered to pre-sexually active young people [46, 47]. This could explain why condom use among post-secondary youths was 12% lower than in respondents with higher secondary education given that sexual debut occurs at very young age.

Living arrangements were strongly associated with condom use during sexual intercourse. Sexually active participants who lived with either one or two parents, and those who lived alone, were more likely to use a condom during sexual intercourse, compared with those who lived with other relatives. This suggests that young people living with relatives other than their parents, and those living with no relatives, were more vulnerable to sexually transmitted diseases and unwanted pregnancy because of limited negotiation powers with their sexual partners concerning condom use. Similar findings have been reported in youth studies elsewhere implying that young people living away from their parents are at a greater risk of sexual exploitation and abuse [48, 49].

Our findings suggest that young people knew how and where to access other forms of contraception apart from condoms. This finding may be due to the health policy environment of Uganda, which emphasizes effective control of STIs, and preventing unwanted pregnancies and their consequences. This policy direction is likely related to Uganda’s much acclaimed success in reducing the prevalence of HIV between the 1990s and mid 2015 from 15% to 4.4%, which was itself premised on strong behaviour change campaigns [50, 51]. However female condoms, post-exposure prophylaxis, and abortion services were inaccessible and unaffordable. Unaffordability of contraception services is a likely correlate of Uganda’s 31–40% rate of unwanted pregnancies, which is one of the highest in the world [52, 53]. Unaffordable contraceptive services could be one of the distal determinants of the common occurrence of child abandonment in Kampala [54]. Evidence suggests that teenage school girls who fall pregnant drop out of school [55, 56], a double tragedy that further entrenches them and their offspring into a cycle of poverty due to missed education opportunities. Uganda could benefit from liberal and open access contraception policies to reduce teen pregnancies, adolescent births and child abandonment. Uganda could learn from the policies of countries like Switzerland and Slovenia, where young people have access to a wide range of contraception services, and record very low rates of teen pregnancy, 0.9% and 0.12% respectively [57].

Not surprisingly, 80.6% of the study participants reported their first sexual encounter was consensual, suggesting that most young people are choosing when they make their sexual debut. Therefore, the onus is on parents, guardians and policy makers to accept adolescent sexuality as a fact, and to provide adolescents with comprehensive sex education. Without this realisation, the social expectation that teens are not sexually active—or that those who are will consistently use contraception—remains a fallacy. Notwithstanding the consensual sexual debut, our data suggests a significant rate of non-consensual sexual debut among the vulnerable younger age group, those with a disability, young people living with non-relatives, and those still at school. Sexual debut as early as nine years suggests a highly sexualized environment in which children engage in sex at an early age, often without their consent. Very young children, especially girls, are exposed to risks such as STIs, early pregnancies and obstetric fistula when they attempt to give birth before their bodies mature [58]. Reports suggest that some sex offenders who are wealthy or have ‘connections’ remain unpunished and are in positions where they continue to abuse their victims without repercussions [59]. Furthermore, we noted that 7.4% of the respondents indicated powerlessness to prevent sexual abuse against them. Stronger action-backed policies are required to protect vulnerable young children, the disabled, and those living away from their parents, from sexual abuse. There is an urgent need for accelerated empowerment of young people with skills and information on prevention and avoidance of unwanted sexual attention, and strategies for how to identify potential sexual abusers.

Uganda successfully rolled back HIV infections especially during the 1990s and early 2000s [60]. Evidence suggests that the decline in multi-partner sexual behaviour was one of the correlates of the decline in HIV infections [51, 61]. However our data suggests that more than half of the study respondents had sexual relationships with multiple partners in the past 12 months, a trend that would seem to undermine the government’s efforts in preventing HIV transmissions. Nonetheless, our findings present an opportunity for new and creative prevention approaches that specifically target young urban people in low resource settings. Uganda has one of the youngest and most youthful populations with 31.4% of the 34.9 million Ugandans aged between 10–24 years [62]. Therefore, specific HIV prevention initiatives targeting young people in slum areas are a direct investment in a productive and future AIDS-free workforce.

## Conclusion

This study has explored current sexual practice among young people in a specific part of urban Kampala. Young people’s sexual and reproductive health remains a challenge in Uganda with significant barriers such as inaccessible and unaffordable services. To address these barriers, a comprehensive and harmonised sexual and reproductive health system that is easily accessible, youth friendly and affordable, and which takes into account local socio-cultural contexts is urgently needed. Such a system needs to incorporate robust sexuality education in lower primary schools, where the majority of children are enrolled due to free universal primary education. Additionally, a functional sexual and reproductive health system with adequate resourcing would be of benefit to all Ugandans.

## Supporting Information

S1 Appendix Supporting Information. Baseline questionnaire -Sexual, reproductive health needs, and rights of young people in slum areas of Kampala, Uganda.
(DOC)
