# Supplementary material for: Sexual, Reproductive Health Needs, and Rights of Young People in Slum Areas of Kampala, Uganda: A Cross Sectional Study
Source: PLoS One. 2017 Jan 20;12(1):e0169721. doi: 10.1371/journal.pone.0169721 (PMC5249247; doi:10.1371/journal.pone.0169721)
Supplement: S1 Appendix — Baseline questionnaire -Sexual, reproductive health needs, and rights of young people in slum areas of Kampala, Uganda. (DOC) [file pone.0169721.s001.doc]

**Questionnaire for young people: Interviewer’s administered**

**Suitable for young people aged 13-25 years**

| Date _ _ /_ _/_ _ _ _ | Enumerator’s name_________ Interview start at: _________ Finishes at_________ |
| --- | --- |
| Survey No._______ | Name of Parish being surveyed_________________ |

**Unit being surveyed: 1=**Household 2= school 3=church/mosque 4=Internet café 5=Other pls specify__________

**Number of people living in the unit** ___________________ Number aged 13-15 years____________________

| 1. **When where you born (DD/MM/YY?) _ _ /_ _ /_ _** |
| --- |
| 1. **Are you a _____ (circle one only) 1. Male 2. Female 3. Do not know** |

1. **What is the highest level of schooling that you have completed? (Circle one only)**

| No education | Primary 1 | Primary 2 | Primary 3 | Primary 4 | Primary 5 | Primary 6 | Primary 7 | Senior 1 | Senior 2 |
| --- | --- | --- | --- | --- | --- | --- | --- | --- | --- |
| Senior 3 | Senior 4 | Senior 5 | Senior 6 | Tertiary 1 | Tertiary 2 | Tertiary 3 | Post-grad 1 | Postgrad 2 | Postgrad 3+ |

1. Who do you live with? Choose only one option.

|  | ^1^With my mother and father |
| --- | --- |
|  | ^2^With my mother, but not my father |
|  | ^3^With my father, but not my mother |
|  | ^4^Parents are alive, but left home to live alone |
|  | ^5^My parents are not alive, I live with other family members |
|  | ^6^My parents are not alive, I live with another family who are not relatives |
|  | ^7^My parents are not alive, I live with friends |
|  | ^8^My parents are alive, I live with relatives |

1. What is your main activity? Choose one

| 1. Paid employment (employee on a salary) | 1. Self-employed (Business/Income Generating Activity | 1. School | 1. Unemployed: Volunteer or unpaid work | 1. Unemployed: No structured activity | 1. Other, pls specify_____ |
| --- | --- | --- | --- | --- | --- |

1. **Do you have any disability?**
2. **Young people learn about puberty - I mean the ways in which boys' and girls' bodies change during the teenage years - from many sources. What has been the most source of information for you on this topic? (tick all that apply)**

|  | 1. School teacher |
| --- | --- |
|  | 1. Mother |
|  | 1. Father |
|  | 1. Brother |
|  | 1. Sister |
|  | 1. Other family members |
|  | 1. Friends |
|  | 1. Doctors |
|  | 1. Internet |
|  | 1. Community outreach officer or worker or nurse |
|  | 1. Parent support group |
|  | 1. Books/magazines |
|  | 1. T/V/Films/Videos/Radio |
|  | 1. Other (Specify…………… |

| 1. **Have you ever had sex with someone? For this survey, sex can mean either vaginal or anal penetrative sexual intercourse** |
| --- |

| Yes^1^ |  | No^2^ |  | Don’t Know^3^ |  |
| --- | --- | --- | --- | --- | --- |

**(if no, skip to question 68)**

1. **How old were you when you first had sex?**

Write age in years: __________________ (Leave blank if they don’t want to respond or have never had sex)

| 1. **The last time you had sex, did you use a condom?** | Yes^1^ |  | No^2^ |  | DK^3^ |  |
| --- | --- | --- | --- | --- | --- | --- |

1. **In the last 12 months, how many different people have you had sexual intercourse with? (circle one only)**

**1.** One person **2.** Two **3.**Three **4.** Four or more people **5.** I have not had sex in the last 12 months

1. **In the last 12 months, how much older or younger was/is your most recent sexual partner? (circle only one)**
2. 10 years or older
3. 6-9 years
4. 2-5 years
5. 1 years or younger
6. Don’t know
7. **If someone tried to have sex with you or touch you sexually and you did not want them to, would you be able to stop them? (circle only one)**
8. Definitely could not
9. Probably could not
10. Probably could
11. Definitely could
12. Don’t know
13. Don’t want to respond
14. **Which of the following statements most closely describes your experience the first time you had sexual intercourse? Circle only one answer**
15. I was willing
16. I was persuaded through gifts, money or other favours
17. I was tricked
18. I feared implications of my refusal to have sex with this person
19. I was forced/raped
20. I have never had sex
21. Don’t want to respond
22. **In the past 12 months, which of the following statements most closely describes your experiences with all the sexual relationships you have had? Circle only one answer.**
23. I was willing
24. I was persuaded through gifts, money or other favours
25. I was tricked
26. I feared implications of my refusal to have sex with this person
27. I was forced/raped
28. I have not had sex in the last 12 months
29. Don’t want to respond
30. **Now I have some other questions on sex and reproduction. I will read you some statements. Please tell me whether you think the statement is true, or not true, or whether you don't know. Tick the box next to each statement**

|  | True | Not True | DK |
| --- | --- | --- | --- |
| 1. It is common for young females to be forced to have sexual intercourse against their will by a stranger, a relative or an older person. | 1 | 2 | 3 |
| 1. A girl can confidently suggest to her boyfriend that he uses a condom without fear and hesitation | 1 | 2 | 3 |
| 1. A boy will not respect a girl who agrees to have sex with him but she insists on him using a condom | 1 | 2 | 3 |
| 1. It is sometimes okay for a boy to force a girl to have sex if he loves her, it does not matter whether she has some feeling for him | 1 | 2 | 3 |
| 1. A boy and a girl should have sex before they become engaged to see whether they are suited to each other | 1 | 2 | 3 |
| 1. It is sometimes justifiable for a boy to hit his girlfriend, as long as they love each other | 1 | 2 | 3 |
| 1. If you (female) or your girlfriend (male) became pregnant, you would never contemplate having an abortion | 1 | 2 | 3 |
| 1. It is mainly the woman's responsibility to ensure that contraception is used regularly to prevent unwanted pregnancy and/or diseases | 1 | 2 | 3 |
| 1. It is not important that you should fall in love with someone first before having sex with them. | 1 | 2 | 3 |
| 1. If a situation arose where you have to use a condom, you know where and how to access it easily | 1 | 2 | 3 |
| 1. You would refuse to have sex with someone who is not prepared to use a condom no matter what. | 1 | 2 | 3 |
| 1. In the last 12 months you have visited a health facility or doctor to receive services or information on contraception, pregnancy, abortion or sexually transmitted diseases | 1 | 2 | 3 |
| 1. If you were to undertake an HIV you are confident that you will be given counselling about what would happen if the HIV test result was positive | 1 | 2 | 3 |
| 1. You would be able to safely disclose the result of your HIV test to your partner (if he or she was not there at the time of the test) if you wanted to | 1 | 2 | 3 |
| 1. If you or your partner receive a positive HIV diagnosis, there are HIV treatment, care and support services that you can access in your community | 1 | 2 | 3 |
| 1. If you are planning birth control or delaying pregnancy (e.g. male or female condoms, pills, IUD, or implant ), you know of a place where you can get them | 1 | 2 | 3 |
| 1. If you have a question about sexually transmitted infections or HIV/AIDS, you have somewhere or someone nearby you can go to for help. | 1 | 2 | 3 |
| 1. If you have a question about abortion, you have somewhere or someone nearby you can go to for help. | 1 | 2 | 3 |
| 1. You can make decisions about whether or not, and when, to have children without fear that anyone will, discriminate against you, or act violently towards you. | 1 | 2 | 3 |
| 1. In your community, a woman in a relationship or a married woman is fee from pressure to have sex when she doesn’t want to. | 1 | 2 | 3 |
| 1. In your community, a woman in a relationship or a married woman can confidently say no to sex without fear. | 1 | 2 | 3 |

1. **Are the following HIV and sexual and reproductive health products/services available in your area and affordable?**

|  | **Available (I)** | | **Know how to access them (II)** | |
| --- | --- | --- | --- | --- |
|  | Yes | No | Yes | No |
| 1. Male condoms |  |  |  |  |
| 1. Female condoms |  |  |  |  |
| 1. Other forms of birth control e.g. birth control pills, implant etc. |  |  |  |  |
| 1. Other forms of STI prevention e.g. dental dams, post-exposure prophylaxis |  |  |  |  |
| 1. STI testing and treatment services |  |  |  |  |
| 1. HIV testing and counselling |  |  |  |  |
| 1. HIV treatment (ARVs) |  |  |  |  |
| 1. Pregnancy testing |  |  |  |  |
| 1. Pre-, peri-, and post-natal health care |  |  |  |  |
| 1. Abortion services and counselling |  |  |  |  |
| 1. Training/information on sexual or reproductive |  |  |  |  |
| 1. Health education services e.g. condom use demonstration |  |  |  |  |

1. If given the opportunity to attend a meeting that asked for youth opinions, which of the following youth issues would you prioritize? (Tick all that apply.)

|  | 1. Sexual and reproductive rights |
| --- | --- |
|  | 1. Sexual and reproductive health |
|  | 1. Integration of HIV services with Sexual and reproductive health services |
|  | 1. Gender equality |
|  | 1. Involvement of voiceless in decision-making processes |
|  | 1. Stopping gender-based violence |
|  | 1. HIV prevention |
|  | 1. Preventing traditional harmful practices such as FGM |

1. **Here are some statements about HIV. For each statement try to answer what you really think: yes, no or don’t know. Pls circle the respondent’s answer for each statement**

| **1=Yes** | **2= No** | **3= Don’t know** |
| --- | --- | --- |

| 1. Can the risk of HIV transmission be reduced by having sex with only one uninfected partner who has no other partners? | 1 | 2 | 3 |
| --- | --- | --- | --- |
| 1. Can a person reduce the risk of getting HIV by using a condom every time they have sex? | 1 | 2 | 3 |
| 1. Can a healthy-looking person have HIV? | 1 | 2 | 3 |
| 1. Can a person get HIV from mosquito bites? | 1 | 2 | 3 |
| 1. Can a person get HIV by sharing food with someone who is infected? | 1 | 2 | 3 |
| 1. If a member of your family became sick with the AIDS virus, would you be willing to care for him or her in your household? | 1 | 2 | 3 |
| 1. If you knew that a shopkeeper or food seller had the AIDS virus, would you buy fresh vegetables from them? | 1 | 2 | 3 |
| 1. If a female teacher has the AIDS virus but is not sick, should she be allowed to continue teaching in school? | 1 | 2 | 3 |
| 1. If a member of your family became infected with the AIDS virus, would you want it to remain a secret? | 1 | 2 | 3 |
| 1. Can the virus that causes AIDS be transmitted from a mother to her baby during pregnancy? | 1 | 2 | 3 |
| 1. Can the virus that causes AIDS be transmitted from a mother to her baby during delivery? | 1 | 2 | 3 |
| 1. Can the virus that causes AIDS be transmitted from a mother to her baby by breastfeeding? | 1 | 2 | 3 |
| 1. Is it possible in your community for someone to get a confidential test to find out if they are infected with HIV? i.e. no one will know the result if you don’t want them to know | 1 | 2 | 3 |
| 1. Don’t tell me your result, but have you ever had an HIV test? | 1 | 2 | 3 |
| 1. Don’t tell me your result, but did you get your result? | 1 | 2 | 3 |
| 1. Do you think you are at risk of getting HIV or another sexually transmitted disease? | 1 | 2 | 3 |

1. **Which one of the following do you think prevent people undertaking an HIV/AID test (Tick as many as appropriate)?**

|  | 1. Not being informed, i.e. do not understand or know the importance of HIV/AIDS testing/where to go |
| --- | --- |
|  | 1. Rejection and discrimination by the community and family if the test becomes positive |
|  | 1. Lack of confidentiality by hospital or clinic staff (everybody knows everybody in the community) |
|  | 1. The cost of the test is beyond the reach of many people |
|  | 1. Procedure is embarrassing for both doctor and the person requesting the test |
|  | 1. Lack of support pre and post testing |
|  | 1. Non-availability of testing kit |
|  | 1. Preference for non-testing, better not to know your HIV status |
|  | 1. Other: please specify |

1. **Do you know your health responsibilities?** 1=Yes 2=No (go to Q 78)
2. **What health responsibilities do you know? (Tick all that apply)**
3. Seeking Medical care
4. Responsibility to provide information e.g. name, age, illness history
5. Responsibility to follow treatment instructions
6. Responsibility if you refuse treatment
7. Responsibility to respect others
8. Other(Specify)
9. **Do you know any health Policies of this country?** 1=Yes 2= No (go to Q80)
10. **Which ones do you know? (Tick all that apply)**

|  | 1. Health Sector Strategic plan (2010-2015) |
| --- | --- |
|  | 1. Uganda Health Management Committee handbook |
|  | 1. Patients’ charter. |
|  | 1. Reproductive Health Policy |
|  | 1. Adolescence sexual and reproductive Health Policy |
|  | 1. National health Policy II |
|  | 1. Same sex relationship |
|  | 1. Other(Specify) |
